# Monomeric, Oligomeric, Polymeric, and Supramolecular Cyclodextrins as Catalysts for Green Chemistry

**DOI:** 10.34133/research.0466

**Published:** 2024-09-09

**Authors:** Makoto Komiyama

**Affiliations:** Research Center for Advanced Science and Technology (RCAST), The University of Tokyo, 4-6-1 Komaba, Meguro, Tokyo 153-8904, Japan.

## Abstract

This review comprehensively covers recent developments of cyclodextrin-mediated chemical transformations for green chemistry. These cyclic oligomers of glucose are nontoxic, eco-friendly, and recyclable to accomplish eminent functions in water. Their most important feature is to form inclusion complexes with reactants, intermediates, and/or catalysts. As a result, their cavities serve as sterically restricted and apolar reaction fields to promote the efficiency and selectivity of reactions. Furthermore, unstable reagents and intermediates are protected from undesired side reactions. The scope of their applications has been further widened through covalent or noncovalent modifications. Combinations of them with metal catalysis are especially successful. In terms of these effects, various chemical reactions are achieved with high selectivity and yield so that valuable chemicals are synthesized from multiple components in one-pot reactions. Furthermore, cyclodextrin units are orderly assembled in oligomers and polymers to show their cooperation for advanced properties. Recently, cyclodextrin-based metal–organic frameworks and polyoxometalate–cyclodextrin frameworks have been fabricated and employed for unique applications. Cyclodextrins fulfill many requirements for green chemistry and should make enormous contributions to this growing field.

## Introduction

Recently, “green chemistry” has been attracting much interest as new technology for sustainable growth of world economy without damages to global ecosystems [1–5]. In this concept, required chemical transformations must be accomplished with the optimal atomic economy and energy efficiency without using hazardous substances and solvents. Compared with organic solvents, water is preferable because of its nontoxicity, safety, and eco-friendliness. Eminent catalysts are necessary to achieve various transformations efficiently and selectively under mild conditions. In order to synthesize complicated chemicals from multiple components, one-pot synthesis is far more desirable than stepwise sequential processes. With these efforts, the cost and energy to purify target products and dispose undesired wastes can be minimized. As described in this review, cyclodextrins (CDs) and their derivatives satisfactorily fulfill all these requirements and are highly promising for advanced green chemistry. Furthermore, their remarkable properties are freely improvable in terms of various chemical procedures.

CDs are cyclic oligomers of D-glucose with cylindrical structures and produced by bacteria in nature [6–13]. In industry, they are manufactured by digesting starch with enzymes (CD glycosyltransferases) and supplied at relatively low prices. The internal diameters of the cavities of α-, β-, and γ-CDs (composed of 6, 7, and 8 glucose units) are about 4.5 to 6.0 Å, 6.0 to 8.0 Å, and 8.0 to 9.5 Å, respectively. On one side of the cavity, their primary hydroxyl groups are orderly arranged, and the other side bears the secondary hydroxyl groups. Thus, CDs show satisfactorily high water solubility and biocompatibility. It is noteworthy that the secondary hydroxyl group of a glucose unit forms a hydrogen bond with the secondary hydroxyl groups of the same glucose unit, and they are further consecutively hydrogen bonding with the adjacent glucose units. As a result, intramolecular rings of hydrogen bonds are constructed at the rim of cavity and stabilize the cylindrical structures of CDs. Furthermore, this ring formation enormously increases the acidity of these hydroxyl groups for efficient catalysis (vide infra). The interior of the cavities of CDs is surrounded by the walls composed of a ring of C–H groups, a ring of glycosidic oxygens, and another ring of C–H groups, and thus is apolar in nature. Accordingly, in water, a variety of hydrophobic guest molecules of suitable sizes are preferentially transferred from aqueous phase to the interior of cavity to form inclusion complexes [14–16]. The CD/guest ratio in inclusion complexes is 1:1 in many cases. The molecular size and shape of guest should fit the cavity for stable inclusion, since van der Waals interactions are the major driving forces for the complex formation. The inclusion process is further promoted by the liberation of “high-energy water molecules”, which are poorly hydrogen bonding in the cavity [15]. Thus, water has been almost exclusively employed as the solvent for the applications of CD (dimethyl sulfoxide and *N*,*N*′-dimethylformamide were exceptionally used instead). Most of unique physicochemical and biological properties of CDs are primarily based on their inclusion complex formation and thus are evident in water. Because of these green characters, CDs have been widely applied for a variety of purposes in our daily lives (pharmaceutics, cosmetics, food, biotechnology, medical, agriculture, catalysis, nanotechnology, environmental protection, and many other fields) [17–33].

**Fig. 1.**
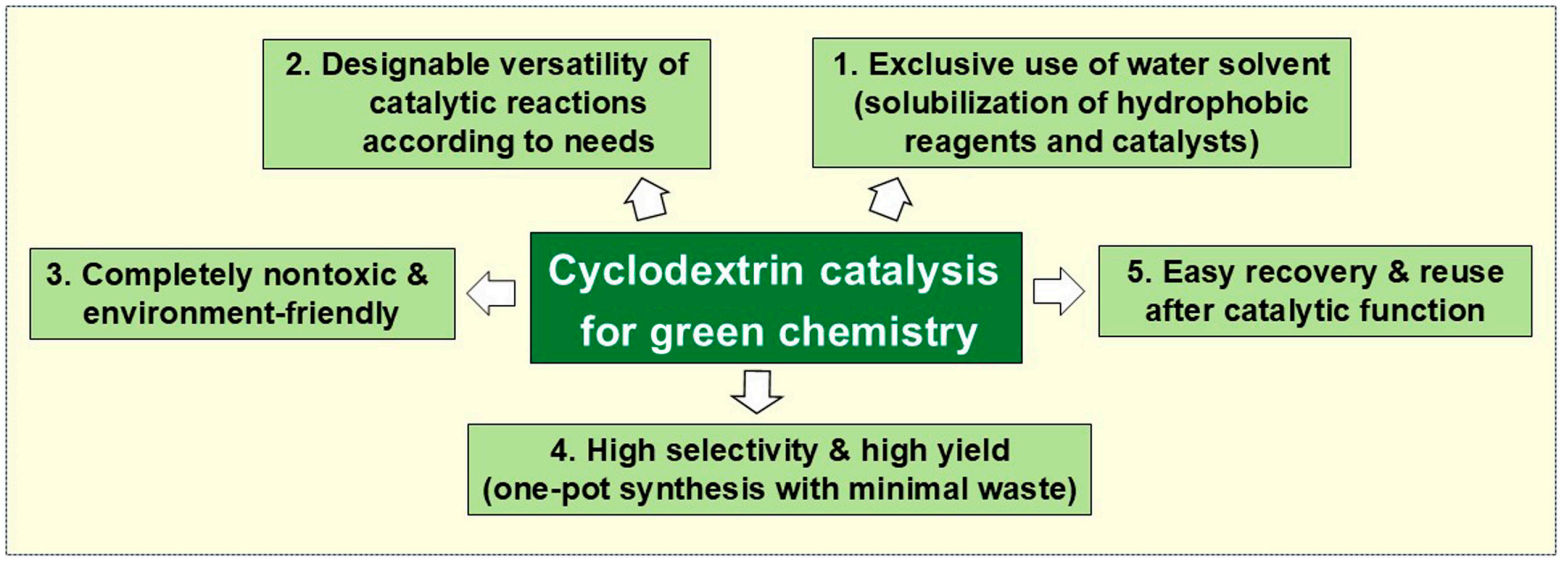
Essential 5 characteristics of CD catalysis for green chemistry.

Among these applications, this review focuses onto CD-mediated chemical transformations. These catalytic processes are featured by 5 characteristics in Fig. 1, all of which are essential for green chemistry [34–36]. First, the reactions are accomplishable in water, as described above. Even hydrophobic reactants and catalysts can be dispersed in water through the inclusion complex formation. Second, CD-catalyzed reactions are wide in scope and freely designable through covalent and noncovalent modifications of CDs [37–43]. When necessary, multiple CD molecules are cooperated for advanced functions. Third, CDs are nontoxic natural products and never pollute environments. Moreover, their release to the environments can be avoided by forming polymers or immobilizing to solid supports. Fourth, the selectivity and the yield of CD-catalyzed reactions are remarkably high so that the desired product is obtainable in high yield with minimal production of wastes. This feature leads to successful one-pot synthesis of complicated chemicals from multiple components. Last, CDs are stable enough to be kept intact during most of chemical transformations and easily recovered and recycled [44]. Sufficiently high chemical stability of CDs under conventional conditions is guaranteed by many literatures. Their deterioration is considerable only under extremely harsh acidic conditions [45]. In summary, all the characteristics of CDs completely fit advanced green chemistry.

In this review, fundamentals on the mechanisms of CD catalysis are briefly described first, mainly for the readers who are not very familiar with this field. Then, the catalysis by monomeric CDs and their chemically modified versions is described. According to the need, multiple CD molecules are assembled to oligomers and polymers to achieve still more advanced catalysis. Combinations with metal catalysis are especially important. Many successful examples of one-pot syntheses are presented. Finally, recently developed CD-based metal–organic frameworks (CD-MOFs) and polyoxometalate (POM)-CD frameworks are described. Throughout this review, mechanistic arguments on the roles of CD for eminent functions are emphasized.

## Basic Mechanisms of CD Catalysis

**Fig. 2.**
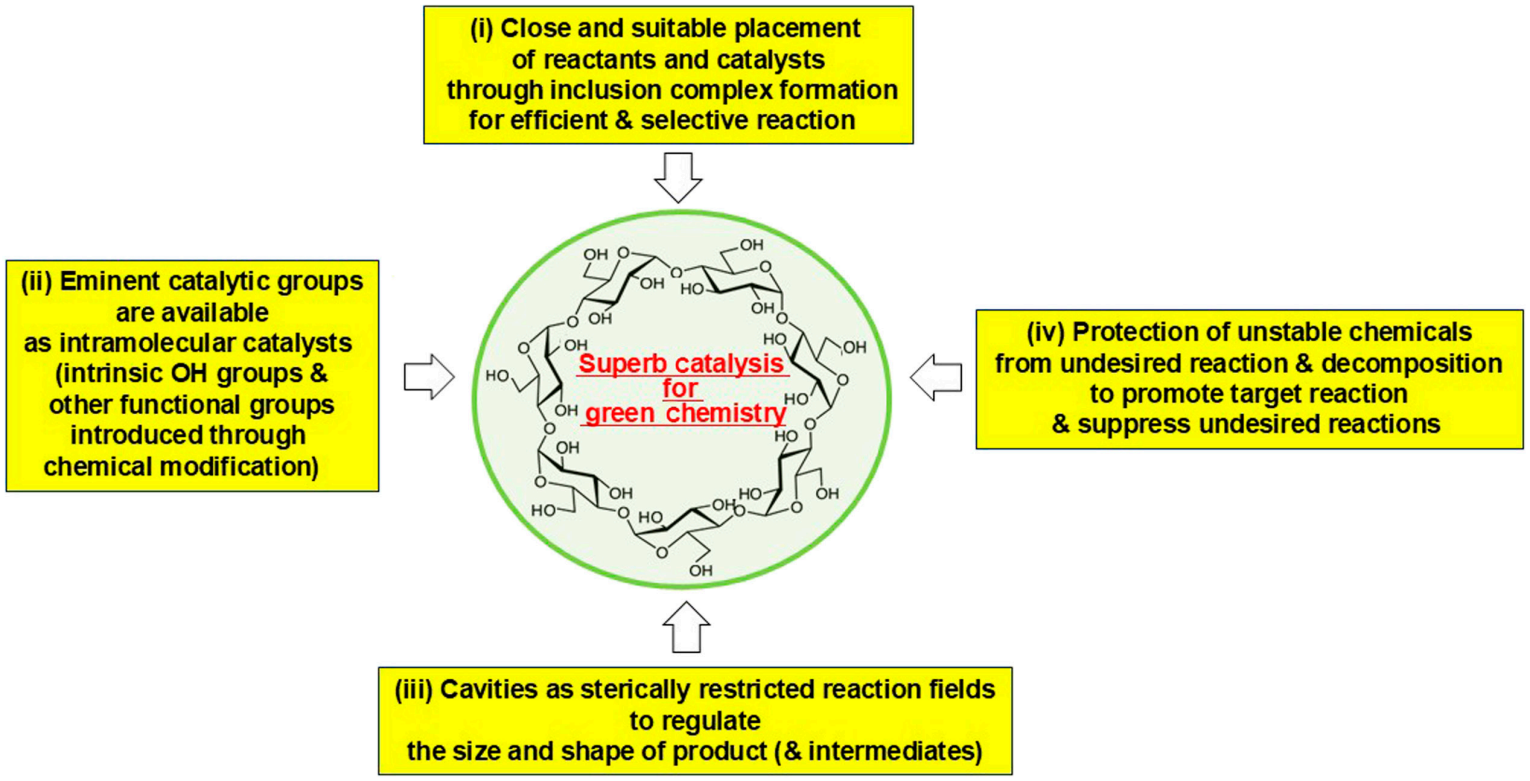
Typical 4 mechanisms of CD catalysis leading to green chemistry. For the purpose of simplicity, only the structure of β-CD is presented.

In the chemical transformations, CDs form inclusion complexes with reactants, intermediates, and/or catalysts in water, leading to the excellent 5 characteristics for green chemistry in Fig. 1. According to previous studies by many laboratories [6–14], these functions of CDs are primarily based on the 4 mechanisms in Fig. 2, which directly reflect the structural and physicochemical features of the inclusion complexes.

a. “Placement of reactants and/or catalysts in close and suitable position”. Through the inclusion complex formation, conventional intermolecular reaction (or intermolecular catalysis) is converted to more efficient pseudo-intramolecular reaction (or catalysis). In addition to these simple proximity effects, the mutual conformations of components are strictly regulated to accomplish high reaction efficiency. These factors are controllable through appropriate chemical modifications of CDs.

b. “Availability of eminent catalytic groups”. The secondary hydroxyl groups of CDs serve as effective intramolecular acid catalysts, since they are highly acidic compared with conventional hydroxyl groups. Their p*K*_a_ (where *K*_a_ is the acid dissociation constant) value is lowered to around 12 by the consecutive formation of intramolecular hydrogen bonds with adjacent glucose units, as described above [6–11]. Accordingly, their alkoxide ions are also abundantly supplied under mild conditions and act as eminent base catalyst. The primary hydroxyl groups function as acid catalyst. Furthermore, desired catalytic groups (either organic or inorganic) are covalently or noncovalently bound to CDs by chemical means and placed at appropriate positions for successful intramolecular cooperations. These chemical modifications almost unlimitedly widen the scope of catalytic reactions.

c. “CD cavities as specific reaction fields”. Stereochemical and physicochemical restraints in the cavity (or its vicinity) induce various specificities in the reaction product (regioselectivity, enantioselectivity, and others). All the products and the intermediates should satisfactorily fit the cavity, since otherwise their removal from the cavity in the course of reactions induces energetic disadvantages to decelerate the reaction. Thus, the sizes of products (and intermediates) and their shapes are strictly controlled by CDs. Furthermore, the microenvironments in the cavities can be suitable for the production of a specific product.

d. “Protection of unstable intermediates (and unstable reactants)”. CDs efficiently protect unstable chemicals in the reaction mixtures. These effects minimize the production of undesired byproducts and waste, and improve the selectivity and the yield of target product. Furthermore, complicated synthesis involving many reactants (and many intermediates) are accomplishable in one-pot manner, even when some of the intermediates and/or products are unstable and otherwise easily decomposed.

In many CD-mediated reactions, multiple factors of these 4 mechanisms (a) to (d) synergistically operate to promote the catalytic activity. Of course, the solubilization of hydrophobic chemicals by CDs in water is important for these mechanisms to work out with sufficient efficiency. Furthermore, some catalytic reactions by CDs involve specific mechanisms, in addition to these typical ones.

## Previous Study Overview

In the early stage of researches, CDs were mainly employed to construct mimics of naturally occurring enzymes on the basis of their inclusion complex formation with substrates [6,7,11]. Artificial models of esterase, peptidase, ribonuclease, and many other enzymes were synthesized [34–50]. As a result, valuable and detailed information on the molecular mechanisms of enzymatic catalysis was obtained. Furthermore, man-made catalysts, which show enzyme-like activity and selectivity, were successfully fabricated.

In addition to the construction of enzyme models, CDs were used as catalysts for organic synthesis to accomplish high product specificity [51,52]. In the 1980s, with the use of β-CD as catalyst, *para*-hydroxybenzaldehyde was synthesized in high selectivity from phenol and chloroform in aqueous alkaline solution [53]. In this Reimer–Tiemann reaction, dichlorocarbene, formed in situ from the chloroform, is the attacking electrophile. Without β-CD, the reaction occurs at both the *ortho*- and *para*-positions of phenol. In the presence of β-CD, however, the *para*-substitution dominantly prevails. Here, dichlorocarbene is accommodated in its apolar cavity, and phenol molecule shallowly enters the cavity from its *para*-carbon side due to its more apolar character. Accordingly, the dichlorocarbene is placed near the *para*-carbon of phenol through these 2 noncovalent interactions and dominantly attacks this carbon for the *para*-selective reaction. By replacing the chloroform in this reaction with carbon tetrachloride, *para*-hydroxybenzoic acid was selectively synthesized with β-CD catalysis [54]. Photo-induced Reimer–Tiemann reaction by β-CD was also *para*-dominant [55].

Later, β-CD was used for selective oxidation of sulfide (R-S-R′) to sulfoxide [R-S(=O)-R′] in water by *N*-bromosuccinimide [56]. Additional oxidation of the sulfoxide product to sulfone [R-S(=O)_2_-R′] was suppressed by β-CD. Similarly, selective synthesis of β-hydroxysulfides from alkenes and thiophenols was catalyzed by β-CD [57]. Beckmann rearrangements of aldoximes to amides [58], Friedel–Crafts alkylation of indoles [59], and Michael addition of thiols [60] were accomplished with high efficiency. Other organic syntheses were also successfully achieved with the use of CD, as reviewed elsewhere [4,34–36,61,62].

## Recent Studies on Catalysis by Unmodified CD

Recent concerns of CD science are focusing onto catalytic synthesis of practically useful chemicals. The concepts of green chemistry have been increasingly emphasized. Many novel successes, including one-pot syntheses of complicated chemicals, were reported. In this section, CDs are employed directly in their native forms without chemical modification.

### Catalysis by unmodified CD without the assistance of metal ion

By using β-CD, 4-(*N,N*-dimethylaminomethyl)phenol was selectively synthesized in water from phenol, formaldehyde, and dimethylamine [63,64]. In this Mannich reaction, an intermediate *N,N*-dimethyliminium [(CH_3_)_2_N^+^=CH_2_] is formed from formaldehyde and dimethylamine and attacks phenol. In the absence of β-CD, both the *para*- and *ortho*-substituted phenols are produced in comparable amounts (53:47 ratio). With the addition of β-CD to the solution, however, the *para*-reaction becomes dominant and the *para*/*ortho* ratio is 90:10 when [β-CD]/[phenol] = 2/1. According to ^1^H-NMR (nuclear magnetic resonance) analysis on the reaction mixture, phenol lies slantingly near the primary hydroxyl side of β-CD cavity. In this outer-sphere complex, the *para*- and *ortho*-positions of phenol are not much differentiated from each other for the attack by *N,N*-dimethyliminium. On the other hand, the final products form conventional inclusion complexes with β-CD. The *para*-product penetrates into the β-CD cavity more deeply than the *ortho*-product and forms more stable inclusion complex. On the basis of this finding, the authors proposed that the β-CD-mediated Mannich reaction is *para*-selective, since the *para*-attack leads to more stable product than the *ortho*-attack. Apparently, this mechanism is different from the mechanism of β-CD-mediated *para*-selective formylation of phenol [53], in which dichlorocarbene is noncovalently placed near the *para*-carbon in inclusion complexes (vide ante). Sufficiently hydrophobic dichlorocarbene forms stable inclusion complex with β-CD for the *para*-selective reaction. However, highly polar *N,N*-dimethyliminium intermediate for the Mannich reaction is hardly accommodated in the cavity of β-CD and attacks the phenol from the aqueous phase. This difference should be responsible for the discrepancy of mechanisms of these 2 CD-mediated *para*-selective reactions. On the other hand, 1,3-dipolar cycloaddition of phenyl nitrone [C_6_H_5_-C=N^+^(CH_3_)-O^−^] with aromatic olefines is catalyzed by γ-CD in water [65]. In its large cavity, these 2 reactants were accommodated and placed in an appropriate position for the cycloaddition.

With the use of CD, the application of amine catalysts for fixation of carbon dioxide was greatly promoted [66]. An amine catalyst 1,8-diazabicyclo[5.4.0]-7-undecene was mixed with 2,3,4,5,6-pentafluorophenolate and β-CD, and the resultant ternary complex was used as catalyst for the cycloaddition of carbon dioxide to epoxides into cyclic carbonates. The reaction smoothly proceeded at 130 °C under 3.0 MPa carbon dioxide pressure. Under these reaction conditions, the phenolate anions were weakly bound by β-CD, and placing the cations of 1,8-diazabicyclo[5.4.0]-7-undecene as counterions outside the cavity. As a result, the cations in aqueous phase efficiently catalyzed the addition of carbon dioxide to the epoxide. Here, the hydroxyl groups of β-CD activated the ring opening of epoxide. Importantly, after the catalysis, the ternary inclusion compound was regenerated by cooling the reaction mixture to room temperature. Thus, the catalyst was easily recovered by simple filtration and reused for the following reaction cycle. The advantages of this system for green chemistry are apparent. Recently, CDs were used to facilitate the recovery of enzyme and its recycling [67]. They also form inclusion complexes with amphiphilic copolymers and regulate the aggregation state of enzyme-containing nanoparticles.

Solubilization of hydrophobic substrates in water by CDs also facilitate various organic synthesis (Friedel–Crafts alkylation, Suzuki–Miyaura cross-coupling, and many other reactions in water) [63,68–76]. Simply by adding catalytic amount of CD, hydrophobic azides and alkynes were solubilized in water, and their click reactions were greatly accelerated [77,78]. Photooxygenation using hydrophobic dyes was promoted by CDs [79]. In pharmacy and medicine, CDs are employed to solubilize water-insoluble drugs [80,81]. For example, remdesivir, an antiviral agent against SARS-CoV-2 (the virus of COVID-19) [82,83], is poorly soluble in water, and thus must be solubilized by CDs for the administration to patients [84]. Acceleration of enzymatic transformations is also ascribed to the solubilization of hydrophobic substrates [85,86]. Interestingly, some chemically modified CDs themselves exhibit intrinsic virus-killing activity [87,88].

### One-pot synthesis by unmodified CD

It is noteworthy that, with the use of CDs as catalysts, various kinds of complicated chemicals can be synthesized from multiple components in one-pot reactions in water. Without CDs, these reactions must be accomplished through stepwise processes, in which the intermediate product of each reaction step is separated from the mixture and purified, and then used for the following step. Otherwise, the final reaction mixtures contain so many compounds that target product cannot be obtained in a sufficient yield and purity. As described above, the catalysis by CDs is featured by very high selectivity and efficiency. The size, shape, and other properties of reaction products (and of intermediate product of each step also) are regulated by steric and physicochemical restrictions of CD cavity. Furthermore, they are protected by CDs from undesired side reactions. Thus, their separation and purification are unnecessary.

**Fig. 3.**
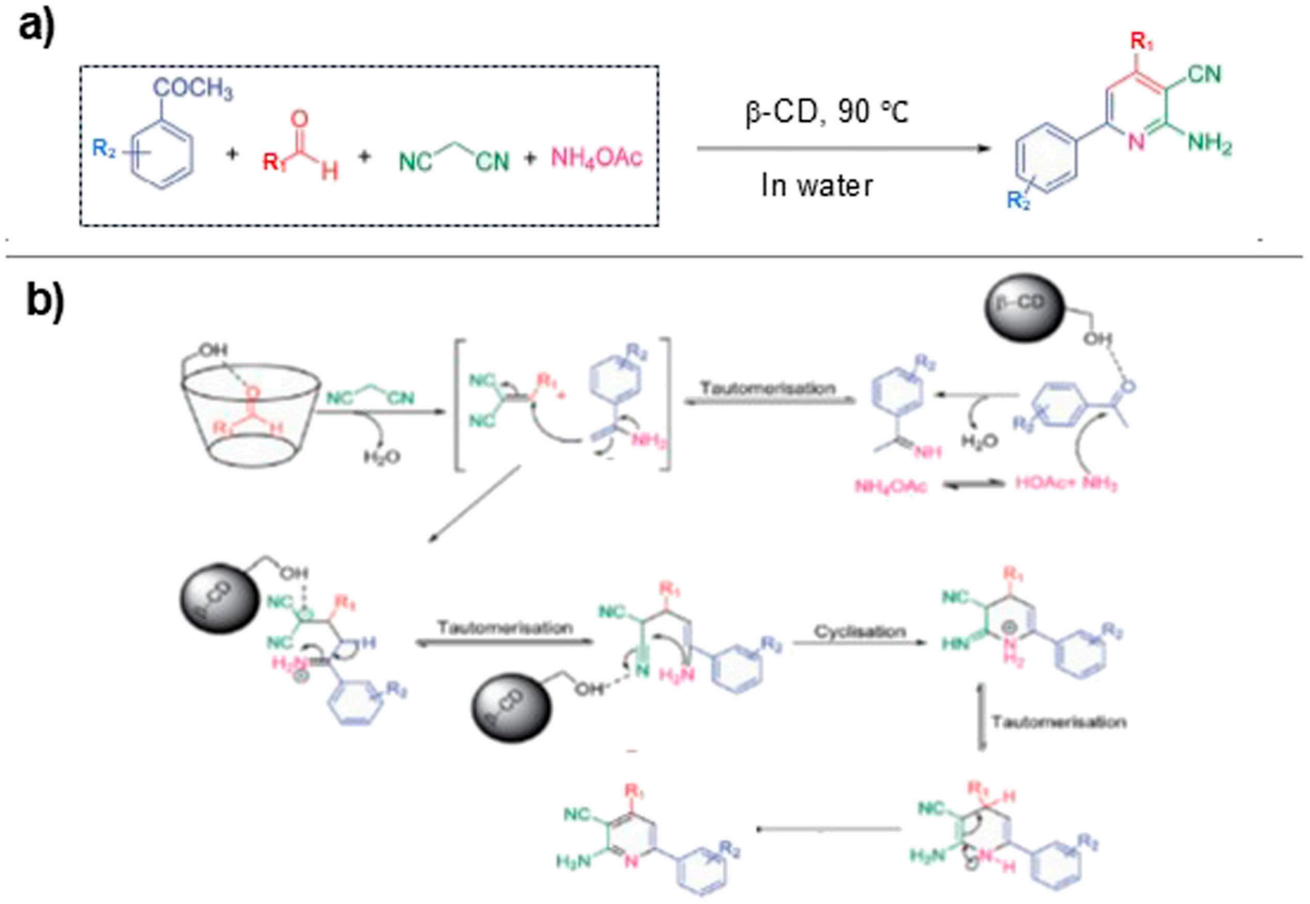
(A and B) Catalysis of β-CD for one-pot synthesis of 2-amino-4,6-diphenylnicotinonitrile from 4 components in water. The acid catalysis by the hydroxyl groups of β-CD promotes the reactions of intermediate products in the cavity. Reproduced with permission from [89]. Copyright 2021 Royal Society of Chemistry.

In Fig. 3, 2-amino-4,6-diphenylnicotinonitriles were synthesized in high yields (>80%) simply by mixing 4 components (aromatic aldehydes, acetophenone, malononitrile, and ammonium acetate) in aqueous solution of β-CD [89]. When the reactions were achieved in the absence of β-CD under the same conditions, however, the final products were never obtained, and only the intermediate products were detected in the reaction mixture. In this one-pot reaction, both aromatic aldehyde and acetophenone are first accommodated in the cavity of β-CD and react with malononitrile and ammonium acetate, respectively (the top row in Fig. 3B). The hydroxyl groups of β-CD aid these reactions as intramolecular acid catalysts. The resultant 2 intermediates further react with each other in the cavity to form their addition product, which is then converted to the next intermediate through a sequence of tautomerization and cyclization (the second row in Fig. 3B). The final intermediate product again tautomerizes to yield 2-amino-4,6-diphenylnicotinonitrile. Apparently, the proximation of 2 reactants (or intermediates) in the cavity, as well as the acid catalysis by β-CD, is essential for this one-pot reaction. The cyclization is also facilitated by the steric effect of the cavity. In a similar way, 2,3-dihydroquinazolin-4(1*H*)-ones were synthesized by one-pot reactions of 3 reagents (aldehydes, isatoic anhydride, and ammonium acetate). With the use of β-CD, many other heteroaromatic compounds were also prepared from multiple components in one-pot fashion in water [67,69,90–98].

All these CD-mediated one-pot syntheses proceed via inclusion of the components in the cavity so that the size of CD is critically important for the success. In fact, a very explicit size effect was observed in one-pot synthesis of *N-*phenylaziridine from aniline, propionaldehyde, and ethyl diazoacetate (aza-Darzens reaction) [99]. When these 3 reagents were mixed in water in the presence of β- or γ-CD, the target product *N-*phenylaziridine was successfully synthesized (30 to 50% yield). γ-CD was more effective than β-CD. Quite interestingly, no product was obtained when α-CD was instead used as the additive. Without the addition of any CD, the synthesis was completely unsuccessful. According to molecular dynamics simulations, a short-lived complex (lifetime = 1.75 ns) is formed from aniline, propionaldehyde, ethyl diazoacetate, and γ-CD. The cavity of γ-CD is large enough to accommodate all the 3 reagents simultaneously to form the 4-component complex. The smaller cavity of β-CD is less preferable for this purpose. On the other hand, the cavity of α-CD is too small to form the 4-component complex, and thus, this CD is completely inactive for the one-pot synthesis.

Compared with conventional thermal reactions, sonochemical reactions are generally more suitable to save energy and reduce hazardous wastes [100–104]. By combining ultrasound irradiation with CD catalysis, eco-friendly one-pot synthesis of pyrazolopyranopyrimidines (anti-inflammatory agents) from 4 components was accomplished in water [105]. In an aqueous solution of β-CD, all the 4 components (aromatic aldehydes, ethyl acetoacetate, hydrazine hydrate, and barbituric acid) were mixed, and ultrasound was irradiated at 50 °C. With this simple procedure, the target product was efficiently obtained (isolated yield > 90%). Both α- and γ-CDs were also effective. Without CDs, however, no product was obtained. This selective synthesis involves a cascade of condensation, addition, and cyclization. An intermediate is in situ synthesized by the condensation of ethyl acetoacetate with hydrazine hydrate, whereas another intermediate is formed by Knoevenagel condensation of aromatic aldehyde and barbituric acid. Then, these 2 intermediates undergo Michael addition with the assistance of acid catalysis by the hydroxyl groups of β-CD, and the resultant adduct leads to the final product through the intramolecular cyclization. Using ultrasonic irradiation in the presence of β-CD in water, 2-amino-4*H*-pyranoquinolines were also synthesized in one-pot fashion from aromatic aldehydes, ethyl cyanoacetate (or malononitrile), and 8-hydroxyquinoline [106].

### Catalysis by the combinations of unmodified CDs with metal ions

**Fig. 4.**
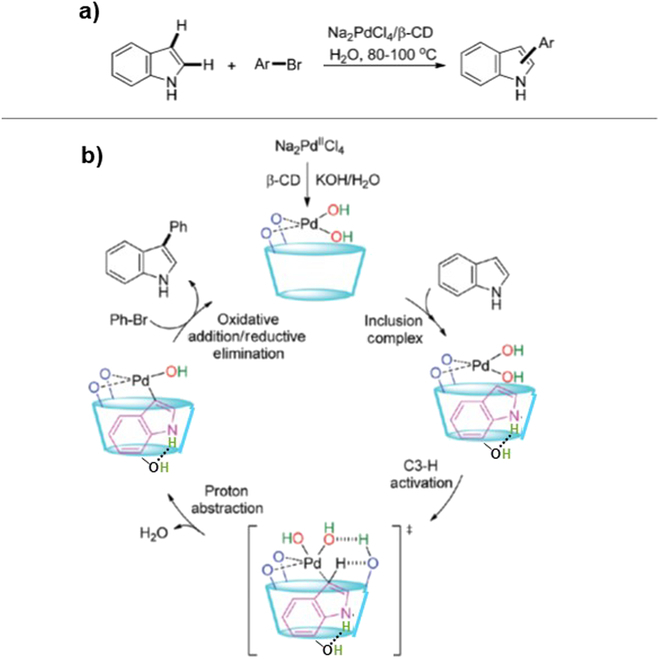
(A) Regioselective C3 arylation of indole by using Na_2_PdCl_4_ and KOH base in the presence of β-CD, and (B) the proposed reaction mechanism for the C3-selective arylation. The catalytic Pd(II) is strongly bound by the secondary hydroxyl groups of β-CD, which are mostly ionized in highly alkaline KOH solution and placed near the C3-H of indole bound by β-CD. Reproduced with permission from [109]. Copyright 2021 Royal Society of Chemistry.

By combining CDs with metal ion catalysts, the scope of catalytic reactions can be greatly widened [107,108]. In most of these approaches, metal ligands are covalently bound to CDs via appropriate linkers (vide infra). When CDs directly act as the ligands to bind metal ions, however, selective synthesis is achievable even without covalent attachment of ligands. In Fig. 4, unmodified β-CD was added to aqueous solution of Na_2_PdCl_4_ (1:1 molar ratio), together with KOH (2 mM) as base catalyst [109]. In the mixture, a β-CD–Pd(II) complex was in situ formed and efficiently catalyzed selective arylation of indole at the 3-position by phenyl bromide. With the use of 5 mol% of β-CD, 3-phenylindol was produced in 91% yield in 2 h (the main byproduct was 2-phenyindole in 7% yield). Phosphine ligands, which are usually employed for these Pd-mediated coupling reactions, were unnecessary. Without the addition of β-CD, however, the reaction was far less selective and much slower. In a longer reaction time (24 h), 3-phenylindol was produced in only 28% yield with 2-phenyindole in 5% yield. When tris(3-sulfonatophenyl) phosphine was added to the solution (in place of β-CD), the reaction was almost nonselective (the yields of 3-phenylindol and 2-phenylindol in 24 h were 39 and 38%, respectively). The substrate scope was broad, and functional-group tolerance was satisfactory. In this catalysis by the β-CD–Pd(II) system, indole is solubilized in water through the formation of inclusion complex (Fig. 4B). The essential point is that Pd(II) ion is strongly bound by the secondary hydroxyl groups of β-CD, since these groups (p*K*_a_ ≃ 12; vide ante) are ionized to alkoxide ions in highly alkaline solutions (2 mM KOH). Thus, the use of toxic phosphine ligands is unnecessary. In the inclusion complex of indole with β-CD, its NH is hydrogen bonding with the primary hydroxyl groups of β-CD (the dotted line in the bottom of cavity), and the C3-H of indole is placed near Pd(II), which is bound by the secondary alkoxide ions. This structure induces preferential activation of C3 position for the site-selective arylation. Very interestingly, this C3 regioselectivity of β-CD catalysis was evidently switched to C2 regioselectivity (preferential production of 2-phenylindole), simply by changing the base catalyst from KOH (2 mM) to KOAc (2 mM). With the use of β-CD and Na_2_PdCl_4_ in the KOAc solution, the 2-phenylindole/3-phenylindole ratio in the product was 85:10.

This remarkable switching of regioselectivity is interpretable in terms of the pH difference of these 2 reaction mixtures (and the resultant difference in the structures of inclusion complexes). The aqueous KOH solution is strongly alkaline (pH > 14), whereas the KOAc solution is only mildly alkaline (pH ≃ 9.5). Accordingly, in the KOAc solution, the secondary hydroxyl groups of β-CD (p*K*_a_ ≃ 12) are in their neutral forms and thus cannot bind Pd(II) with sufficient strength. Under these conditions, the metal ion is almost freely moving around in the aqueous phase. Furthermore, these hydroxyl groups stably form an intramolecular ring of hydrogen bonds so that β-CD takes the canonical cylindrical structure to provide a cavity of its intrinsic size (6 to 8 Å diameter). There, indole must include the cavity perpendicularly (with its long axis parallel to the longitudinal axis of the cavity), not horizontally as in Fig. 4B. Its benzene ring is accommodated in the cavity, and the 5-membered heterocycle is placed near the rim. In this structure, the C3 position of indole is protected by the wall of cavity from the activation by the Pd(II) ion, which mostly exists in aqueous phase. As a result, the arylation dominantly proceeds at the C2 position, which remains unprotected from the wall of β-CD, exactly as was observed. In the KOH solution (pH > 14), however, the secondary hydroxyl groups of β-CD are mostly dissociated, and the intramolecular ring of hydrogen bonds is broken. Accordingly, β-CD takes a “flexibly opened structure”, in which the cavity is somewhat distorted and expanded to accommodate large guest molecules. Into this enlarged cavity, indole can horizontally include, as depicted in Fig. 4B. Under these conditions, the reaction is successfully C3 selective with the catalysis by the Pd(II) ion, which is fixed by the secondary alkoxide ions.

Catalytically active Pd(II)–β-CD complex for Suzuki–Miyaura cross-coupling reactions was prepared by mixing β-CD with Pd(OAc)_2_ in NaOH solution [110]. Alternatively, Pd(OAc)_2_ was mixed with β-CD, choline bromide, and 2,6-diaminopyridine [111]. A bimetallic catalytic site of phosphatase was mimicked by using γ-CD [112]. Two Zn(II) complexes were connected by a long flexible aliphatic linker. Only in the presence of γ-CD, these 2 Zn(II) complexes were fixed in close proximity through the inclusion of the linker portion to γ-CD and showed sufficient bimetallic cooperation for the hydrolysis of phosphoesters.

### Dispersion of metal nanoparticles in water by unmodified CDs

Metal nanoparticles are very active catalysts for various kinds of transformations, but their poor solubility in water often hampers wide applications to green chemistry [113,114]. CDs can satisfactorily solve these problems. For example, CeO_2_ nanoparticles, which are highly active for the hydrolysis of phosphoesters, were solubilized by CD in water [115]. To aqueous solution of CeNO_3_ (10 mM), β-CD (0.25 mM) was added. After sonication for 1 h, NaOH was introduced under vigorous stirring, and the mixture was heated to 110 °C. The resultant solutions rapidly decomposed paraoxon (a neurotoxic organophosphorus pesticide).

For the applications to catalysis, drug delivery, bioimaging, and other purposes, Au nanoparticles were dispersed by using β-CD [116–118]. Their sizes were successfully controlled by seed-mediated growth method [119]. First, Au nanoparticles as core seeds were prepared by heating β-CD and HAuCl_4_ (10 mM each) to 100 °C in phosphate buffer (pH 7.0) [120]. Then, different volume of this seed solution was added to buffer solution containing β-CD and HAuCl_4_ (10 mM each), and the mixtures were refluxed for 1 to 2 h. With this double-step procedure, the size of Au nanoparticles was successfully controlled to predetermined values. Hydrothermal procedure is also available for nanoparticle preparation [121]. CDs effectively controlled the size and the shape of mesoporous silica nanoparticles in surfactant-templated and NaOH-catalyzed silica condensation [122]. By using a boron cluster as both Au(III)-reducing agent and γ-CD-binding molecule, monodispersed Au nanoparticles were immobilized on the surface of Fe_3_O_4_ [123].

## Catalysis by Chemically Modified CD

**Fig. 5.**
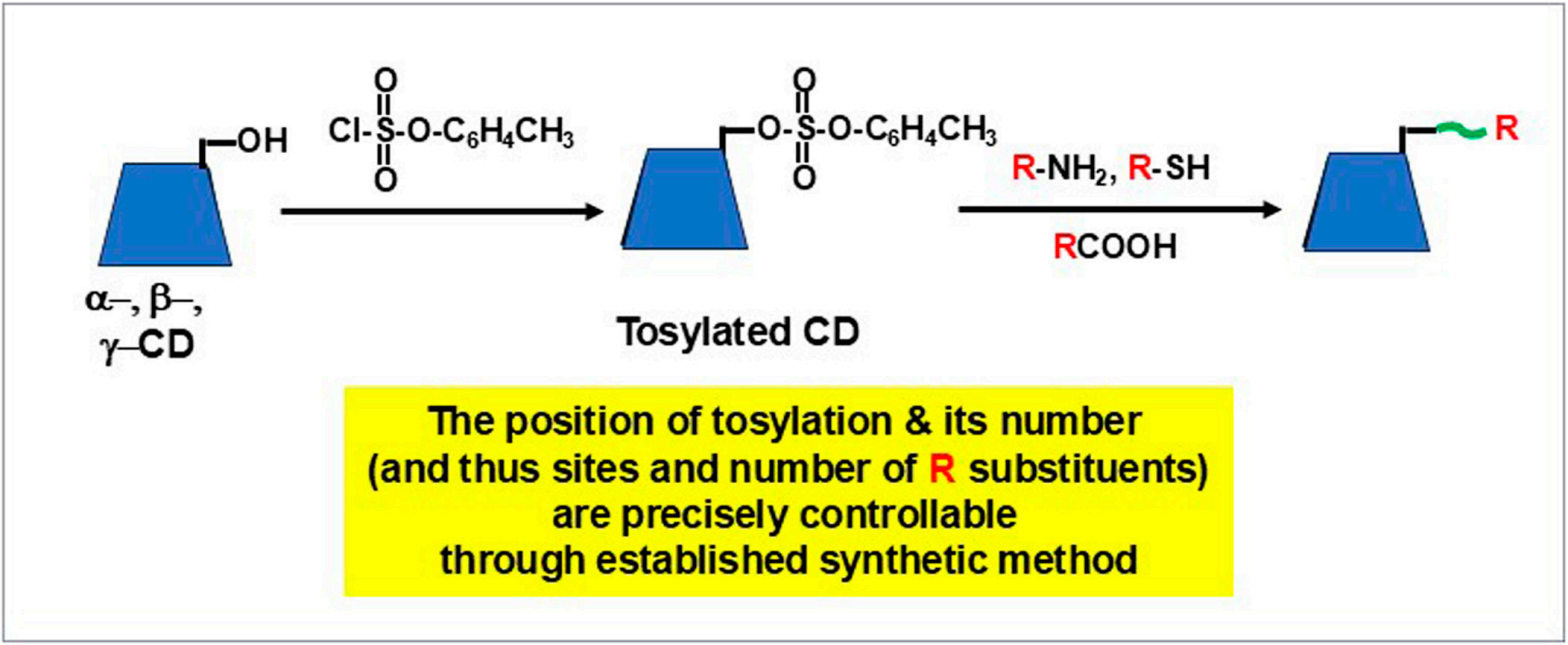
Chemical modification of CD through tosylation of predetermined hydroxyl group(s). The substituent R (red) is bound by the linker portion (green) produced by the nucleophilic attack toward the tosylated CD. Note that the required number of functional group(s) can be introduced to desired site(s) of CD.

One of the most important characteristics of CDs is their easy and precise chemical modifications. With the use of firmly established synthetic methods, required functional group(s) can be straightforwardly introduced to desired sites [38,40,43]. In a typical method, target hydroxyl group(s) is activated by the tosylation with p-toluenesulfonyl chloride and then converted to desired functional groups through nucleophilic substitution (Fig. 5). The position and number of these substituents can be precisely controlled in terms of the structure of tosylated CDs. Solid-phase synthetic approaches [124], as well as mechanochemical approaches [125], are also available for the modification of CDs. In 1994, β-CD bearing 2 imidazole groups was employed for intramolecular aldol condensation, and its efficient catalysis was ascribed to acid/base cooperation of imidazolium ion and neutral imidazole [126]. Promising roles of chemically modified CDs for organic synthesis were indicated.

Currently, various chemically modified CDs (e.g., 2-hydroxypropyl-CDs and methylated CDs) are manufactured in industry and commercially available at reasonable prices [127]. Each of these CD derivatives shows unique physicochemical and biological functions. However, it should be noted that the numbers and positions of substituents on these CD derivatives are strongly dependent on their preparation methods (and thus on the commercial sources). Accordingly, appropriate items should be carefully chosen from the commercial lists to accomplish desired performances.

### Catalysis by modified CD without the assistanceof metal ion

In per-6-amino-β-CD, all the primary hydroxyl groups of β-CD are altered to amino groups [128]. In the presence of this modified β-CD, Henry addition of nitromethane to benzaldehyde rapidly proceeded with very high enantiomeric excess (99%) [129]. Unmodified β-CD is inactive for the catalysis. In the present selective synthesis, benzaldehyde is included in the cavity of per-6-amino-β-CD, together with nitromethane, as confirmed by electrospray ionization–mass spectrometry. Importantly, its aldehyde residue is located near the primary hydroxyl side of the cavity due to the hydrogen bonding with the amino group (probably in its protonated form). Note that both neutral amino groups and protonated ammonium ions are abundant in the reaction mixture, since the p*K*_a_ values of these amino groups widely distribute from 8.9 to 6.5. Then, the methyl group of nitromethane is activated by the intramolecular base catalysis of amino groups, and the nucleophilic attack of its C atom toward the benzaldehyde is promoted. In this reaction, the attack by the nitromethane necessarily occurs from the primary hydroxyl rim side, which bears the amino groups, leading to the dominant formation of the (*R*)-isomer. After completion of the reaction, the product was extracted with ethyl acetate, and the recovered per-6-amino-β-CD was reused for the following run without detectable deterioration.

**Fig. 6.**
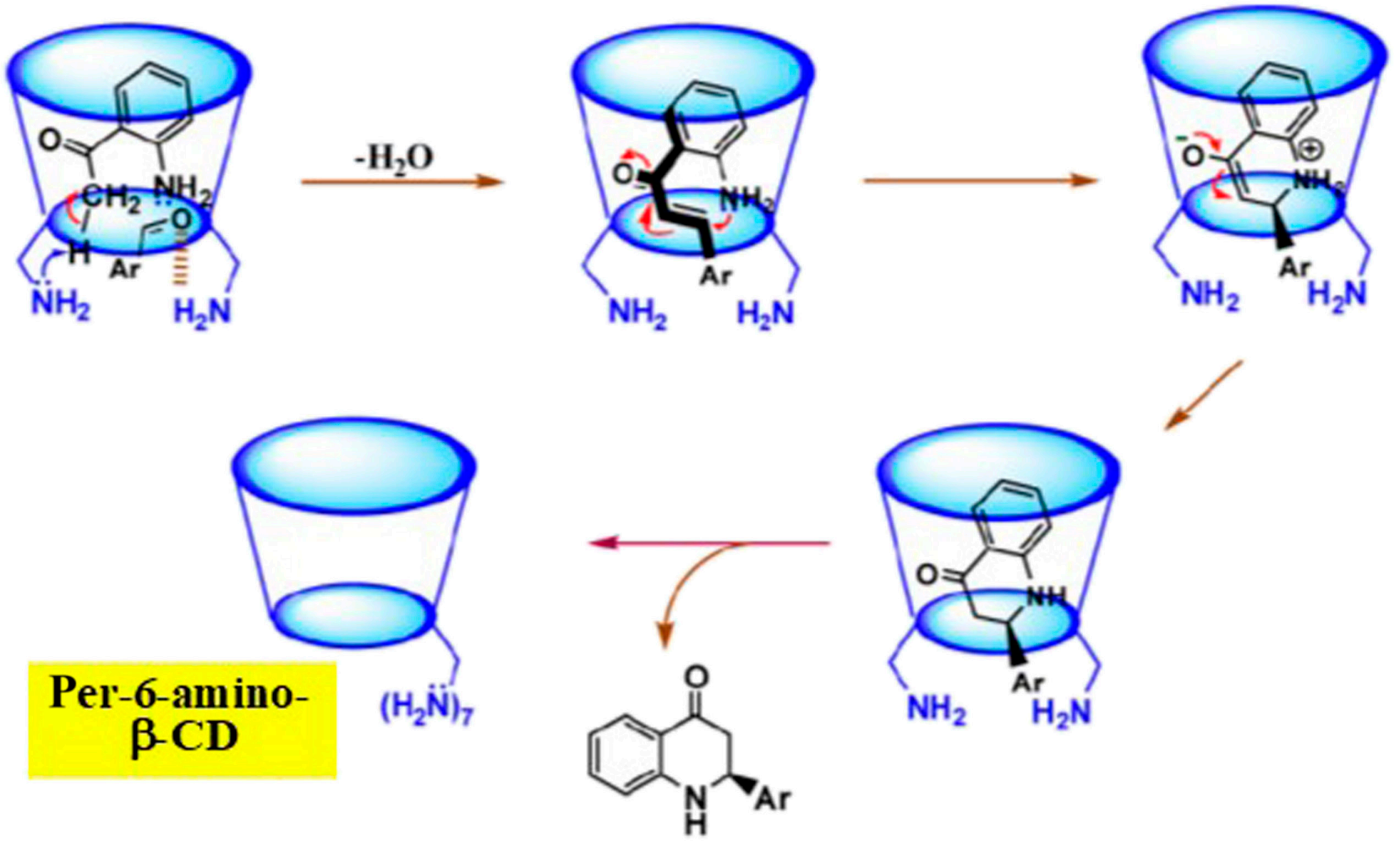
The catalysis of per-6-amino-β-CD for asymmetric synthesis of 2-aryldihydroquinolone from *o*-aminoacetophenone and arylaldehyde. Reproduced with permission from [130]. Copyright 2013 American Chemical Society.

In Fig. 6, per-6-amino-β-CD was employed for asymmetric synthesis of 2-aryldihydroquinolone from *o*-aminoacetophenone and arylaldehyde, which involves a sequence of several chemical transformations [130]. The first step is the formation of *o*-aminochalcone by the nucleophilic attack of *o*-aminoacetophenone toward arylaldehyde in the cavity. This step is promoted by the intramolecular base catalysis of the primary amino groups of the modified β-CD (top left). Simultaneously, another amino group (probably in its protonated state) activates the arylaldehyde by acid catalysis. Since the nucleophilic attack proceeds in the amino-functionalized narrow rim side, high enantioselectivity is accomplished (99% enantiomeric excess), exactly as described above for Henry addition. In the following step, the chalcone cyclizes via nucleophilic attack of the 2′-amino group to the β-carbon (top middle), followed by tautomerization to 2-aryl-2,3-dihydro-4-quinolone (bottom).

Conjugates of proline with β-CD were effective for aldol condensation between aldehyde and acetone [131,132]. For asymmetric reduction of methyl benzoylformate, β-CD was conjugated with (1*S*,2*S*)-(+)-1,2-diaminocyclohexane [133]. Sulfonic acid-functionalized β-CD was effective as acid catalyst [134–138]. On the other hand, methyl-β-CD promoted through the solubilization of water-insoluble reagents the pyrrole synthesis [139], Rh-catalyzed hydroformylation (production of aldehyde from alkene with hydrogen and carbon monoxide) [140,141], and asymmetric preparations in microorganism [142]. 2-Hydroxypropyl-β-CD was also effective [143–145]. It is noteworthy here that some one-pot syntheses by modified CDs can be achieved even under solvent-free conditions. For example, 2-amino-4*H*-benzo[b]pyran derivatives were successfully synthesized without the use of water, simply by grinding per-6-amino-β-CD with aromatic aldehyde, 1,3-cyclohexanedione, and malononitrile [146]. The reaction was very rapid, and attained 95% conversion for only 1 min with the use of 9 mol% catalyst. In aqueous solution of per-6-amino-β-CD, however, much longer reaction time (>1 h) was required. For solvent-free one-pot synthesis of 1,2,4,5-tetrasubstituted imidazole derivatives from 1,2-diketones, aryl aldehydes, ammonium acetate, and aromatic amines, β-CD bearing propyl sulfonic acid was effective [147]. Solvent-free one-pot synthesis by unmodified CD was also reported [148].

### Catalysis by metal complexes of chemically modified CD

By attaching appropriate ligand(s) to CDs through chemical modification, a variety of metal complexes of discrete structures can be bound to predetermined sites of CDs [149]. For example, thymine-modified β-CD was synthesized via tosylation of one of the primary hydroxyl groups and straightforwardly converted to the Pd(II) complex by adding Pd(OAc)_2_ [150]. This catalyst was very effective for Suzuki–Miyaura couplings in water. Pyridinium-bearing β-CD and ethylenediamine-bearing β-CD were also used to prepare Pd(II) catalysts for coupling and hydrogenation [151–154]. For hydroformylation [155–157], the Rh complex was conjugated to β-CD-based Schiff base ligands and others, whereas the Ru complex was bound to β-CD for ring-opening metathesis polymerization [158,159]. A conjugate of Mn porphyrin with α-CD epoxidized *cis*-polybutadiene with *trans*-epoxide preference [160]. For azide-alkyne cycloaddition (click reaction), the Cu(I) complex of thiosemicarbazide- or quinolinium-functionalized β-CD was effective [161,162]. By a conjugate of the Pd(II) complex with β-CD, a dodecapeptide was selectively hydrolyzed at the X-Pro linkage adjacent to a Phe residue [163,164]. For enantioselective oxidization of glucose, α-CDs were modified with 6 imidazolium ions and combined with citrate-stabilized Au nanoparticles to cover the surface of nanoparticles [165,166]. Among 2 enantiomers of glucose, L-isomer preferentially enters the cavity of α-CD and contacts with the Au nanoparticles for the conversion to L-gluconate. A number of CD-conjugated metal complexes were reported for various reactions, as previously reviewed [108].

**Fig. 7.**
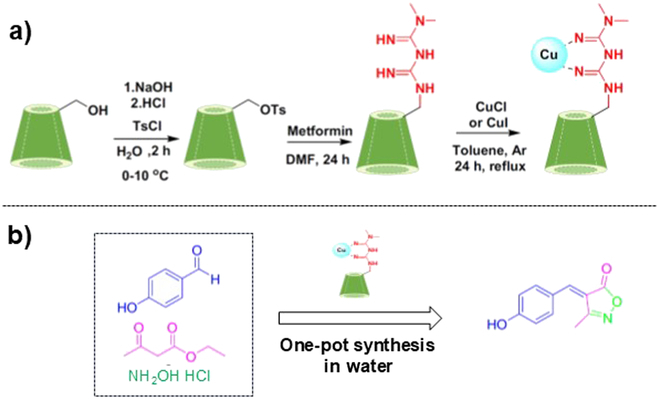
(A) Procedures to modify β-CD with metformin–Cu(I) complex, and (B) one-pot synthesis of an isoxazole derivative using this conjugate. Cu(I) acts as acid catalyst to activate both ethyl acetoacetate and benzaldehyde in different steps for the efficient synthesis. Reproduced from [167] under a Creative Commons Attribution 4.0 International License.

In Fig. 7, a metformin (red part) was connected to the primary hydroxyl side of β-CD, and its Cu(I) complex was used for one-pot synthesis of isoxazole derivative from 4-hydroxybenzaldehyde, ethyl acetoacetate, and hydroxylamine hydrochloride [167]. With the addition of only 2 wt% catalyst, the target product was obtained almost quantitatively (97% yield) in 3.6 min. Without Cu(I), the reaction hardly occurred. In the first step of this one-pot synthesis, the Cu(I) complex activates ethyl acetoacetate by withdrawing the electrons from the carbonyl group of acetyl group as a Lewis acid catalyst. As a result, the nucleophilic attack of the amino group of hydroxylamine toward this carbonyl carbon is promoted. An oxime is produced and then converted to isoxazol-5-one by the cyclization through steric restriction of β-CD cavity. Then, the carbanion at the C4 carbon of this intermediate attacks the carbonyl carbon of benzaldehyde to generate the target product. In this second step, the benzaldehyde is included in the β-CD cavity, and the Cu(I) complex again functions as intramolecular acid catalyst to promote the electrophilicity of its carbonyl carbon.

The conjugates of metal complex with CDs are easily recovered from reaction mixtures and repeatedly used. For example, magnetite nanoparticles, prepared by co-precipitation of FeCl_3_ and FeCl_2_ with ammonia, were treated with 3-aminopropyltriethoxysilane and then reacted with the Pd(II) complex of monotosyl-β-CD. The β-CD–Pd(II) complex was covalently connected to the magnetite nanoparticles and recovered from the reaction mixtures by using an external magnet [168]. Alternatively, various Cu(II) complexes of β-CD or its derivative were connected to magnetic nanoparticles using appropriate linkers [169,170]. Attaching of metal–CD complexes to silica gel was also useful to obtain immobilized catalysts [171–173].

### Precise positioning of metal ions on CDs for highly specific functions

**Fig. 8.**
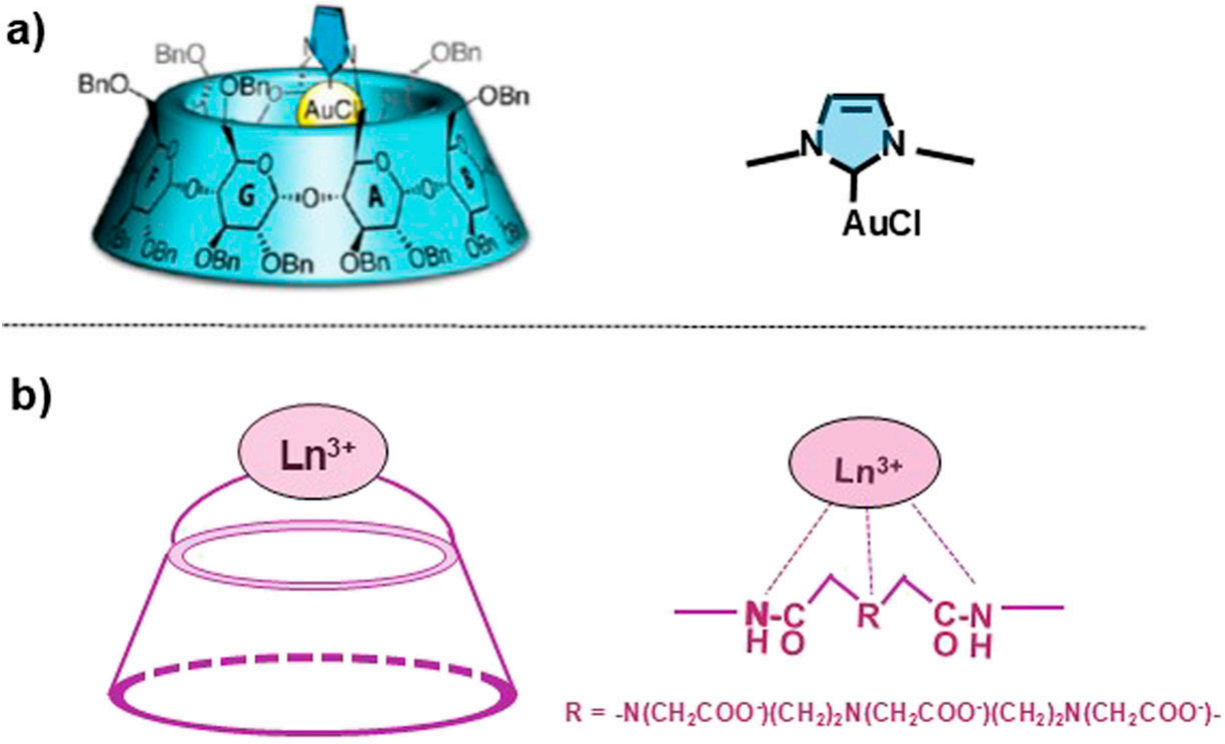
Precise positioning of metal ions in CD conjugates. The modes of metal binding are shown in the right-hand sides. (A) (NHC-capped β-CD)/Au complex in which Au(I) ion is rigidly fixed inside the cavity of β-CD from the primary hydroxyl side. Reproduced with permission from [182]. Copyright 2020 American Chemical Society. (B) Externally capped β-CD in which a lanthanide ion is fixed in the outside of the cavity at the primary hydroxyl side.

In order to control CD-mediated chemical transformations more precisely, metal ions should be rigidly immobilized at predetermined positions of CD molecule, rather than simple connection of them to CD by conventional linkers. For this purpose, an N-heterocyclic carbene (NHC; cyclic carbene with 2 neighboring nitrogen atoms) is highly appropriate, since this ligand is a strong sigma donor to regulate both the steric and electronic properties of metal ions [174]. For example, the NHC–Au(I) complex is formed from imidazole and AuCl (the right-hand side of Fig. 8A). Through the conjugation of this complex to permethylated β-CD using a conventional linker, enantioselective cycloisomerizations were carried out [175–177]. Note that, in spite of the presence of many methyl groups, per-methylated β-CD is sufficiently water-soluble, mainly because the ring of intramolecular hydrogen bonds of the secondary hydroxyl groups in native β-CD (vide ante) is broken to make the whole molecule flexible and dynamic.

In Fig. 8A, this methodology was further extended, and metal ions were rigidly fixed within the cavity of CD by an NHC ligand [178–182]. The NHC ligand is stiffly connected to a pair of glucose units A and D of CD through 2 covalent linkages so that its metal-binding site is precisely orienting toward the inside of the cavity of CD. The synthetic procedure is straightforward. All the hydroxyl groups of α- or β-CD are first benzylated (Bn), and 2 of these benzyl groups are removed from the primary hydroxyl groups of glucose units A and D. Then, these 2 hydroxyl groups are activated by mesylation and reacted with imidazole to bridge the 2 primary hydroxyl groups of A and D units with an NHC group. Finally, Ag_2_O is added to provide the (NHC-capped CD)/Ag complex, in which Ag(I) is rigidly fixed in the cavity of perbenzylated CD from the primary hydroxyl side. Similarly, the Cu(I) and Au(I) complexes are obtained through either direct treatment of NHC-capped CD with the corresponding metal salt or the transmetalation of the (NHC-capped CD)/Ag complex. Encapsulation of metal ions in the cavity was confirmed by NMR analysis. These metal complexes showed enormously high enantioselectivity for hydration and lactonization in water, since the catalytic function of metal center is strictly restricted to the inside of the cavity of CD. In the cycloisomerization of 1,6-enyne by the (NHC-capped β-CD)/Au complex, for example, the enantiomeric excess was as high as 98%.

These (NHC-capped CD)/M complexes induce high regioselectivity, and more interestingly, the regioselectivity switches depending on the cavity size [181–183]. In the borylation of phenylacetylene by bis(pinacolato)diboron [(Bpin)_2_], for example, the (NHC-capped β-CD)/Cu complex showed 91% selectivity for the formation of the branched product [C_6_H_5_-C(Bpin)=CH_2_]. However, the (NHC-capped α-CD)/Cu complex showed reversed regioselectivity and produced the linear product [C_6_H_5_-CH=CH(Bpin)] in 90% selectivity. Apparently, the size of the cavity (α-CD versus β-CD) induces the distinctive regioselectivity switch. The borylation reactions proceed via the binding of Bpin to Cu(I). In the (NHC-capped β-CD)/Cu complex, the cavity of β-CD is large enough to form a pit next to the metallic center. Thus, the phenylacetylene substrate can be included in the cavity with the alkyne residue first from the secondary hydroxyl side, and the borylation reaction by the Cu(I) ion can occur there to provide the branched product. In the α-CD complex, however, the small cavity is tightly occupied by the metal center, and no space is available for the phenylacetylene to be accommodated. The linear product is produced through the approach of phenylacetylene to the Cu(I)-bound Bpin with perpendicular orientation to the Cu–B bond. Consistently, the (NHC-capped α-CD)/Cu(I) complex preferentially provided linear (*E*)-vinyl boron isomers in the methylborylation of terminal alkynes by (Bpin)_2_ and CH_3_I [184]. A dinuclear complex (NHC)Cu-FeCp(CO)_2_ (Cp = cyclopentadienyl) was also encapsulated in the cavity of CD [185].

In another type of metal-capped CD, one side of the cavity (the primary hydroxyl side) was covered by metal complexes (Fig. 8B) [186]. A significant difference from the (NHC-capped CD)/M complexes in Fig. 8A is that the metal complexes are located outside the cavity, and thus, the whole cavity is kept vacant and available for inclusion complex formation of other guest molecules. For this type of modification of CD, the primary hydroxyl groups of 2 glucose units of β-CD (A and D units) were first replaced by amino groups, which were then reacted with diethylenetriaminepentaacetic dianhydride. As a result, the primary hydroxyl side of the cavity was capped by the corresponding ligand, which strongly binds a variety of metal ions. These metal complexes, especially of paramagnetic lanthanide ions, were useful to analyze the dynamic motions of inclusion complexes by NMR spectroscopy. These metal-capping approaches should also be highly promising for specific metal-mediated chemical transformations.

### Chemically modified CDs to disperse catalytic metal nanoparticles in water

Amphiphilic CD derivative and carboxylic acid-functionalized CDs were used as additives to reduce metal ions with NaBH_4_ to nanoparticles [187–189]. β-CD-bearing amino residues were effective to embrace Ag nanoparticles [190]. Alternatively, metal nanoparticles were immobilized to the hydrogels formed from β-CD and cellulose [191], or embedded in β-CD-modified polyvinylidene fluoride membranes [192]. Nanoparticles of Pd were accommodated in α-CD-polyurethane nanosponge [193]. Poly(*N*-vinyl-2-pyrrolidone)-stabilized Au nanoparticles, covered with CD derivatives, were effective for asymmetric oxidation [194].

## CD Oligomers

**Fig. 9.**
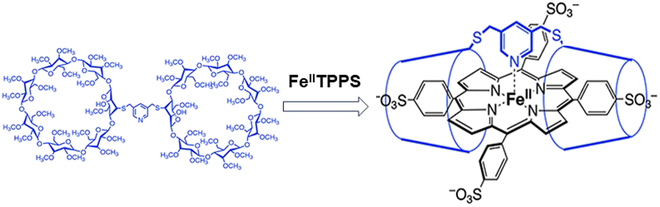
Accommodation of tetrakis(4-sulfonatophenyl)porphinato]iron(II) (Fe^II^TPPS) in a dimer of per-O-methylated β-CD for reversible binding of molecular oxygen in water. The pyridine moiety in the linker functions as the fifth ligand for the O_2_-binding Fe(II) center.

As described above, CDs show superb activities for various chemical transformations. When substrate molecules and/or catalysts are very large, however, the cavities of monomeric CDs are too small to accommodate them sufficiently. One of the solutions to these problems is to construct CD oligomers and assemble multiple CD units in ordered fashion. In Fig. 9, a dimer of per-O-methylated β-CD unit was combined with [tetrakis(4-sulfonatophenyl)porphinato]iron(II) (Fe^II^TPPS) to fabricate an elegant and practically useful mimic of myoglobin (an oxygen-binding protein) [195–198]. In myoglobin, the active center (iron porphyrin) is placed in hydrophobic pocket of the protein and protected from irreversible deactivation in the O_2_ binding–release cycle [199]. In order to mimic this hydrophobic pocket of the protein, a dimer of permethylated β-CD was prepared by connecting the secondary hydroxyl groups of 2 β-CD units with a linker. Furthermore, a pyridine moiety was incorporated to the linker to provide the fifth ligand of the O_2_-binding Fe(II) (left in Fig. 9). In water, this β-CD dimer formed a stable 1:1 complex with Fe^II^TPPS, in which 2 sulfonatophenyl groups of Fe^II^TPPS were included into the cavities of 2 per-O-methylated β-CD units. As a result, the Fe(II) complex was embraced in a large hydrophobic space, which was constructed by the apolar cavities of 2 β-CDs (right in Fig. 9). The multiple methyl groups on the rims of β-CDs further promote the effect. Exactly as designed, this myoglobin mimic formed a chemically and thermodynamically stable adduct with molecular oxygen in aqueous solutions. Reversible binding–release of O_2_ was successfully repeated in water (pH 7.0) at 25 °C without measurable deterioration. This is the first myoglobin mimic that satisfactorily works in water and is applicable to clinical purposes. In addition to reversible binding of O_2_, this artificial receptor also strongly bound carbon monoxide and was employed to remove CO from blood by ligand exchange [200–202]. Furthermore, the active site of cytochrome c oxidase (a heme/copper hetero-binuclear complex to reduce molecular oxygen) was mimicked by introducing a Cu(I) catalyst (for the reduction of O_2_) to this O_2_ binder [203]. To the linker portion in the per-O-methylated β-CD dimer, a terpyridyl-Cu(I) was incorporated and placed near the Fe(II) ion. In an aqueous solution at pH 7 and 25 °C, this supramolecular system rapidly reacted with O_2_ to form a superoxo-type complex (Fe^III^-O_2_^−^/Cu^I^), mimicking the key step in the reduction of O_2_ to water by the naturally occurring enzyme.

With the use of cooperation of 2 (or more) CD molecules, large-sized fluorescent dye molecules were trapped, and their spontaneous aggregation in water was suppressed to provide unique photo-emitting systems [204]. In order to stably accommodate a pyrene in isolated apolar space, a [3]rotaxane was constructed [205]. In this rotaxane, 2 permethylated α-CD molecules were piling each other with the secondary hydroxyl sides (all methylated) inward, whereas the pyrene functions as the axle component. Accordingly, the pyrene was successfully isolated from other pyrene molecules in the solutions and emitted only its monomeric fluorescence (blue light emission). In order to synthesize these photo-emitting rotaxane structures, cucurbit[6]uril (a synthetic host molecule of cyclic structure [206]) is very convenient as the third component, since it enormously accelerates the alkyne-azido coupling reactions [207–209]. Two alkyne groups are bound to a pyrene dye, and an azido group is connected to a stopper molecule. When the pyrene derivative, the stopper derivative, and hexakis(2,3-di-*O*-methyl)-α-CD are incubated at 25 °C in the presence of cucurbit[6]uril, the click reactions proceed very smoothly even without the use of Cu(I) as cocatalyst [210]. By this simple procedure, the fluorescent dye is accommodated in the dimer of the α-CD derivative in the rotaxane, which is capped by the cucurbit[6]uril/stopper complex at both ends. By piling 2 γ-CD molecules in rotaxane structures, in place of 2 α-CDs, 2 molecules of large fluorescent dyes (e.g., pyrene and perylene) can be simultaneously included in the γ-CD dimer [211,212]. Upon photoirradiation, these 2 dye molecules in the spatially restricted large cavities construct asymmetrically twisted excimers, emitting circularly polarized luminescence.

For azide-alkyne cycloaddition (click reaction) in water, a ligand containing 3 β-CD units was combined with Cu(I) ion [213]. The ligand was synthesized by reacting azido-bearing β-CD with tripropargylamine in the presence of CuSO_4_ and sodium ascorbate (click reaction) [214]. For biological applications, CuSO_4_ was added to this ligand, and Cu(II) was reduced in situ to Cu(I) with sodium ascorbate. The 3 β-CD units in the catalyst can solubilize hydrophobic azide and/or alkyne reagents in water, and further place them in proximity through inclusion complex formation for efficient coupling. The turnover number was as high as 45,000 at 20 ppm (parts per million) concentration. Furthermore, 2 CD units were connected by appropriate linkers to promote various chemical reactions through their cooperation [215–219].

## CD Polymers

### Polymeric CD catalysts

There are a number of methods to synthesize CD-based polymers of desired structures [34,220–222]. The most significant advantage of these polymeric catalysts is easy and successful recovery for repeated use, as expected. Moreover, the cooperation of multiple CD units induces unique functions that are otherwise unachievable. For example, crosslinked β-CD polymers, prepared by crosslinking 1,6-hexamethylamine-functionalized β-CD with glutaraldehyde, are eminent catalysts for Henry condensation and Knoevenagel condensation through acid/base catalytic cooperation [223]. Crosslinked β-CD polymers were also prepared by click reaction between per-(6-azido-6-deoxy)-β-CD and 1,4-diethynylbenzene [224]. Other crosslinking agents of CDs easily available are diisocyanates [225], 1,1′-carbonyldiimidazole [226], and epichlorohydrin [227].

Furthermore, various catalysts or functional chemicals were covalently or noncovalently bound to CD polymers and employed for pharmaceutics, medicines, and others [12,77,228–231]. Catenanes involving CDs show various unique properties [232–236]. In order to improve the performance of CD polymers, molecular imprinting technique is useful and effective [237,238]. In the presence of a molecule of choice, CDs are crosslinked so that the structure of this molecule and its physicochemical property are memorized by the crosslinked CD polymer in terms of mutual conformations of multiple CD units. This method is especially useful to recognize large-sized guest molecules, which are hardly trapped by one CD molecule.

### Dispersion of metal nanoparticles in water

CD polymers successfully disperse catalytically active metal nanoparticles in aqueous solutions [239–241]. CD polymers are usually more effective than monomeric CDs because of the cooperation of multiple CD units. Moreover, these immobilized systems allow easy recovery of valuable metal catalysts without loss. In the presence of crosslinked β-CD polymers, NaAuCl_4_ was reduced by NaBH_4_ to Au nanoparticles for various transformations [242]. Ag nanoparticles were also bound to CD polymers [243,244]. Pd nanoparticles were immobilized in the beads, which were prepared from β-CD, diphenyl carbonate, sodium alginate, and CaCl_2_ [245]. Bimetallic nanoparticles were also immobilized in CD polymers [246]. To the solution of β-CD, Pd(OAc)_2_ and CuCl (1:1 ratio) were added together with hexamethylene diisocyanate and incubated at 60 °C. Nanoparticles of mixed alloys Cu*_x_*Pd*_y_* were formed in the polymeric catalysts to show enhanced activity. On the other hand, colloidal Fe nanoparticles, prepared by reducing FeCl_3_ with NaBH_4_ in the absence of CD, were immobilized to β-CD polymers [247]. Fe_3_O_4_ nanoparticles were also available [248]. Pd nanoparticles were conjugated with Cu/Al layered double hydroxide [249] or graphene oxide [250]. The scope of applications and designs of these approaches is almost unlimited.

## Supramolecular Assemblies of CDs

Under appropriate conditions, CDs are spontaneously assembled in ordered fashion through noncovalent interactions. The structures of these assemblies are controllable in terms of molecular design. In the resultant supramolecules, mutual orientations of multiple CDs are strictly regulated to construct new large-sized vacant spaces. These supramolecules should be promising for advanced green chemistry.

### CD-based metal–organic frameworks

**Fig. 10.**
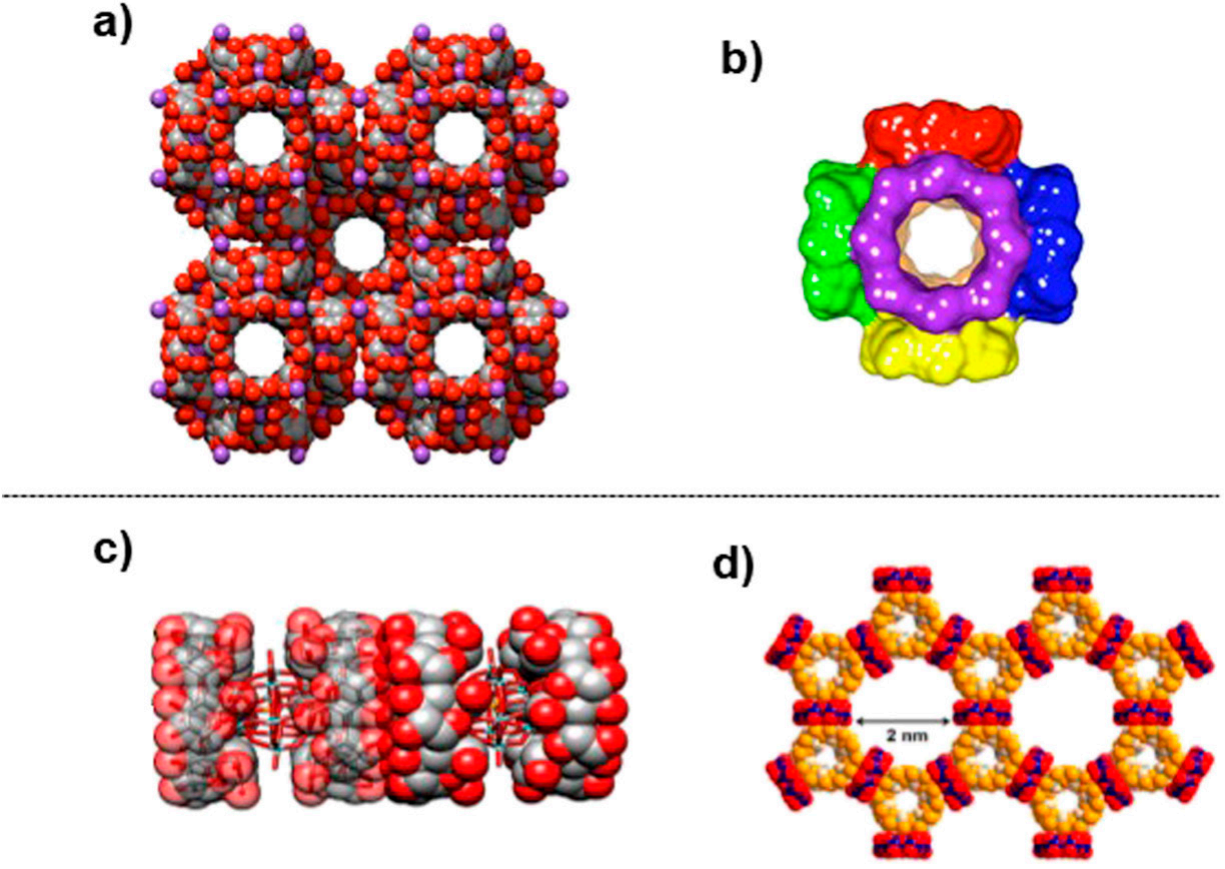
Supramolecules constructed from CDs. (A) Extended structure of CD-MOF (K^+^ ion) formed by body-centered packing of the cubic (γ-CD)_6_ repeating motif as the unit cell. (B) The unit cell (3.1-nm edge) of CD-MOF, in which the primary faces of the γ-CD units point inward and the secondary faces of the γ-CD units point outward. Reproduced with permission from [254]. Copyright 2021 American Chemical Society. (C) Supramolecule of [PMo_12_O_40_]^3−^ POM (stick) with γ-CD (space-filling), in which the POM forms inclusion complex with γ-CD. Reproduced with permission from [263]. Copyright 2015 American Chemical Society. (D) Supramolecule of [AlMo_6_O_18_(OH)_6_]^3−^ POM (red/dark blue) with α-CD (orange), in which the POM is located outside the cavity of α-CD without forming inclusion complex. Reproduced with permission from [283]. Copyright 2022 American Chemical Society.

CD-MOFs are composed of γ-CD and alkali metal cations [251–254]. In water, γ-CD and KOH are mixed in 1:8 ratio, followed by slow diffusion of the vapor of MeOH (or EtOH) over a period of several days. CD-MOF is obtained as colorless cubic crystals (200 to 400 μm size). In this supramolecular framework, cubic (γ-CD)_6_ repeating motif is the unit cell (3.1 nm edge), in which the primary hydroxide faces of γ-CD units point inward with the secondary hydroxide faces outward (Fig. 10A and B). Extended body-centered frameworks of these (γ-CD)_6_ units contain spherical pores that are interconnected by alkali metal cations to form both cylindrical and triangular channels. By replacing KOH with RbOH or CsOH in the preparation procedure, the CD-MOF is also obtainable. On the other hand, Li^+^ can be incorporated by crystallizing γ-CD with LiOH/KOH mixture [255]. Other improved preparation methods are also available [254]. Thin films of CD-MOFs can be fabricated on glass (or silica) substrate by epitaxial growth method [256]. Chemically modified pyrenes are first anchored on the substrate, and γ-CD was bound through pyrene/γ-CD interaction. The resultant γ-CD-functionalized substrate is immersed in aqueous solution of γ-CD and K_2_CO_3_, followed by diffusion of MeOH vapor. By employing 4-methoxysalicylate anions as the secondary building block together with γ-CD, hybrid frameworks of more complicated structures are also synthesized [257].

In order to prepare this CD-MOF, γ-CD must be employed. When α-CD was treated with RbOH in the same manner, a fiber-like crystal was produced [258]. Here, α-CD molecules form porous and left-handed helical channels, which are interdigitated with each other. A similar result was obtained with the use of KOH, in place of RbOH [259]. On the other hand, β-CD-based MOF was prepared by adding appropriate additive (e.g., 1,2,3-triazole-4,5-dicarboxylic acid and 4-toluenesulfonic acid) as template for crystallization [260]. Apparently, the C_8_-symmetry of γ-CD is essential to construct the supramolecule in Fig. 10A and B.

As expected, CD-MOF (of γ-CD) shows enormously specific catalysis in chemical transformations. For example, its crystal was immersed in 1-anthracenecarboxylate solution for 14 days and then irradiated with ultraviolet (UV) light for [4+4] photocycloaddition [261]. The major product was the anti-head-to-head isomer of 60% enantiomeric excess. In this supramolecule, the substrate molecules were accommodated inside the tunnels between spherical cavities, and the cycloaddition proceeded in this specific reaction field with regio- and enantioselectivity. When this photoreaction was carried out in aqueous solutions of native γ-CD, however, the cycloaddition occurred in the cavity of one γ-CD molecule and preferentially produced the anti-head-to-tail isomer. Furthermore, unique metal nanoparticles were prepared by reducing metal salts within CD-MOF [262,263]. In a typical procedure, CD-MOF crystals were immersed in acetonitrile solution of AgNO_3_ or HAuCl_4_, where the metal ions were reduced by the hydroxide counterions in the CD-MOF. The resultant crystals were dissolved in water to provide Ag (or Au) nanoparticles, which were loaded onto catalytic supports. By dissolving 2 different kinds of metal salts in acetonitrile in the preparation step, bimetallic nanoparticles were also synthesized [264]. The roles of CD-MOFs as nanoreactors for various selective reactions are also remarkable [261,265–267]. Upon the irradiation of CD-MOF with 2-benzoylbenzoate, only 1 of the 2 C–H bonds on the C6 of a glucose unit was selectively substituted with a phenylisobenzofuranone group [268]. All other C–H bonds of glucose were kept intact, since 2-benzoylbenzoate was aligned inside the (γ-CD)_2_ tunnels in CD-MOF to preclude their functionalization. Alternatively, CD-MOF efficiently catalyzed electrochemical NH_3_ synthesis through enrichment of nitrate near the cathode by hydrogen-bonding interaction and electrostatic interaction [269]. Moreover, these CD-MOF supramolecules have been widely applied to various purposes (e.g., separation, sensing, electrical memory, and drug delivery) [270–278]. Their applications should be still more widened by increasing the durability in solvents, for example, through covalent linkages of the γ-CD units.

### POM-CD frameworks

CDs also form supramolecules with POMs, which are all-inorganic metal–oxygen clusters of discrete structures and effectively catalyze various reactions in water [279,280]. A POM-CD framework is straightforwardly prepared by incubating γ-CD (or β-CD) with H_3_PMo_12_O_40_ in water [281]. The γ-CD–POM complex takes a 2:1 sandwich-type structure, wherein each [PMo_12_O_40_]^3−^ trianion is encapsulated by the primary faces of 2 γ-CD molecules (Fig. 10C). This 2:1 complex is further lined up longitudinally through [O-H···O] interactions in a one-dimensional (1D) columnar fashion. Another POM [XW_11_MO_40_]^*n*−^ (X = P or Si and M = Mo^V/VI^ or V^IV/V^) also forms supramolecule with γ-CD through inclusion complex formation [282]. In other types of frameworks, POMs (e.g., [AlMo_6_O_18_(OH)_6_]^3−^) are located outside the cavity of CDs, without inclusion complex formation (Fig. 10D) [283]. The POM/α-CD ratio was 3:4 with honeycomb structure, whereas the POM/γ-CD ratio was 1:1 with checkerboard-like structure. The composite of SiW_12_ with γ-CD effectively catalyzed the epoxidation of alkene [284]. In order to oxidize alcohol, alkene, and thiophene, [(β-CD)_3_(SiW_12_O_40_)] was employed as phase-transfer catalyst [285], whereas POM/α-CD frameworks of porous structures were effective for sulfoxidation of sulfides [286].

Furthermore, an advanced green catalyst was fabricated by employing 3D-printing technology to place β-CD units in 3D frameworks as binding sites of Wells–Dawson POM [X_2_M_18_O_62_]^*n*−^ (M = W, Mo; X = P, Si) [287]. In water, this POM was suspended with β-CD nanosponge, prepared by using diphenyl carbonate as crosslinking agent, and stirred at 70 °C for 24 h. After being dried in vacuo, the resultant powder was mixed with epoxyacrylate resin and subjected to a 3D printer, followed by curing with UV light. The POM was fixed in a desired framework to catalyze Knoevenagel condensation of aldehyde with malononitrile in water with high activity and recyclability. Alternatively, γ-CD oligomer, obtained by using 1,6-hexamethylene diisocyanate as crosslinker, was combined with Keggin-type POMs to provide supramolecular crosslinked hydrogels, which showed reversible sol–gel phase transitions responding to redox or thermal stimuli [288].

One of the most important applications of POM-CD frameworks is the improvements of the performance of rechargeable lithium–sulfur battery, which is a promising candidate for the next-generation energy storage due to high energy density, nonhazardous nature, and low cost [289,290]. This battery uses lithium metal as the anode and sulfur composite as the cathode. The half-reaction on the anode is Li ⇄ Li^+^ + e^−^, whereas the one on the cathode is S + 2Li^+^ + 2e^−^ ⇄ Li_2_S. The reaction in the cathode side is actually more complicated and involves the formation of lithium polysulfides (Li_2_S*_x_*, 3 ≤ *x* ≤ 8), which are soluble in liquid electrolyte [291]. These polysulfides diffuse to the anode side and irreversibly deposit on the lithium anode, resulting in poor recharge cycles of this battery (shuttle effect). In order to alleviate this effect, CDs have been employed to trap the lithium polysulfides through supramolecular complex formation and suppress their diffusion to the anode. Grafting of β-CD polymers onto the cathode is effective [292,293]. Furthermore, β-CDs serve as efficient diffusion channel of Li^+^ ions [293–295]. The cavity size of β-CD is suitable to accommodate solvated Li^+^ ion. On the basis of these 2 findings, a POM–β-CD supramolecular framework was synthesized through self-assembly of a Krebs-type POM ([Zn_2_(WO_2_)_2_(SbW_9_O_33_)_2_]^10−^) and β-CD, and used as a separator of lithium–sulfur battery [296]. In this POM/β-CD assembly, 2 β-CDs and one POM are bridged by Na^+^ cations without inclusion complex formation. A slurry of this POM/β-CD framework, carbon nanotube, and conductive carbon black was prepared and deposited on the surface of a polypropylene separator to form a thin layer of 4-μm thickness. The Li–S buttery involving this modified separator exhibited remarkably high electrochemical performances. The lithium polysulfides are efficiently captured by host–guest interactions with β-CD to suppress shuttle effect, whereas Li^+^ ion promptly diffuses through the supramolecular channel of β-CD. Furthermore, the POM catalyzes the interconversion between the lithium polysulfides and Li_2_S [297]. The supramolecule prepared from Keggin-type H_3_PW_12_O_40_ and γ-CD was also employed for a similar purpose [298]. In order to suppress the shuttle effect still more efficiently, CD polymers showing higher polysulfide-trapping activity are necessary. One of the most promising methods is to prepare CD polymers through molecular imprinting technique (vide ante). By polymerizing CDs in the presence of the polysulfides, the mutual conformations of multiple CD residues in the polymers are regulated to bind the polysulfides cooperatively and thus effectively. The use of mixtures of α-, β-, and γ-CDs (or their derivatives) in this molecular imprinting process could be effective, since the CD units of different guest-binding properties, placed at appropriate positions in the polymers, can show synergetic binding.

## Conclusions and Prospects

As described in this review, CDs show remarkable catalytic performances in a wide variety of organic syntheses. It is especially noteworthy that all the syntheses can be (and should be) carried out in water, since inclusion complex formation of CDs requires water solvent as the medium. Moreover, CD promotes the water solubility of hydrophobic chemicals. Accordingly, the CD-mediated catalytic reactions proceed in (or near) the cavities of CDs and thus are accomplished with high selectivity and high yields. The production of undesired wastes is kept to the minimum. These factors lead to many successful one-pot syntheses from multiple components. Very importantly, these remarkable features of CD catalysis can be further improved through chemical procedures (covalent or noncovalent introduction of functional groups, combination with water-insoluble catalysts, construction of supramolecules, and many others). Molecular design of these modified systems is highly free and almost unlimited. CDs are obtainable in large scale at reasonable prices and nontoxic, environment-friendly, and recyclable. Moreover, deep eutectic solvents have been recently prepared by combining CDs (or their derivatives) with other hydrogen-bonding components and attracting much interest as eco-friendly green solvents (recent reviews [299–301]). Applications of CDs to pharmaceuticals, food, agriculture, environmental protection, and many other fields are also highly attractive. These advanced catalysts should be essential for further developments of green chemistry. Undoubtedly, CDs should be one of the most promising players for further developments of these growing and urgent fields.

An important goal of relevant studies should be to fabricate highly active and selective catalysts for still more complicated chemical transformations. In almost all previous studies, target products of chemical transformations (and the reagents used) have been limited to rather small molecules, which can be accommodated in 1 (or occasionally 2) CD cavities. Accordingly, the number of functional groups in these molecules has also been limited. In the near future, however, our target products should be much larger in size and possess so many functional groups. Precise transformation of these molecules is difficult with the use of conventional catalysts. There, we can develop sophisticated catalysts by assembling a multiple number of modified CDs so that both the cavities of these modified CDs and their functional groups are placed complementarily to the corresponding parts of target molecules. The catalytic groups are also provided by these modified CDs. Note that the kind and the number of functional groups in each of modified CDs are freely designable, and these modified CDs are assembled in an ordered manner through supramolecular science and/or molecular imprinting technique. Combinations of differently modified CDs can also be employed when necessary. As presented in this review, cooperative catalysis by multiple CDs was already evident in some of previously reported oligomeric, polymeric, and supramolecular CD systems. However, the proposed strategy is far more advantageous in that required CD assemblies as the catalysts are precisely obtainable according to detailed molecular design. These approaches have not yet been much attempted, but should be very fruitful in terms of remarkable progresses in the relevant fields.
